# Mobile Manipulators in Industry 4.0: A Review of Developments for Industrial Applications

**DOI:** 10.3390/s23198026

**Published:** 2023-09-22

**Authors:** Nooshin Ghodsian, Khaled Benfriha, Adel Olabi, Varun Gopinath, Aurélien Arnou

**Affiliations:** 1 LCPI, Arts et Métiers Institute of Technology (AMIT), HESAM Université, 75013 Paris, France; 2 LISPEN, Arts et Métiers Institute of Technology (AMIT), HESAM Université, 59046 Lille, France; 3 Volvo Construction Equipment AB, 635 10 Eskilstuna, Sweden; 4 OMRON Industrial Automation, 94130 Nogent-sur-Marne, France

**Keywords:** mobile manipulators, Industry 4.0, human–robot interaction, technology readiness level

## Abstract

In the realm of Industry 4.0, diverse technologies such as AI, Cyber-Physical Systems, IoT, and advanced sensors converge to shape smarter future factories. Mobile manipulators (MMs) are pivotal, fostering flexibility, adaptability, and collaboration in industrial processes. On one hand, MMs offer a remarkable level of flexibility, adaptability, and collaboration in industrial processes, facilitating swift production line changes and efficiency enhancements. On the other hand, their integration into real manufacturing environments requires meticulous considerations, such as safety, human–robot interaction, and cybersecurity. This article delves into MMs’ essential role in achieving Industry 4.0’s automation and adaptability by integrating mobility with manipulation capabilities. The study reviews MMs’ industrial applications and integration into manufacturing systems. The most observed applications are logistics (49%) and manufacturing (33%). As Industry 4.0 advances, the paper emphasizes updating and aligning MMs with the smart factory concept by networks of sensors and the real-time analysis of them, especially for an enhanced human–robot interaction. Another objective is categorizing considerations for MMs’ utilization in Industry 4.0-aligned manufacturing. This review methodically covers a wide range of considerations and evaluates existing solutions. It shows a more comprehensive approach to understanding MMs in Industry 4.0 than previous works. Key focus areas encompass perception, data analysis, connectivity, human–robot interaction, safety, virtualization, and cybersecurity. By bringing together different aspects, this research emphasizes a more integrated view of the role and challenges of MMs in the Industry 4.0 paradigm and provides insights into aspects often overlooked. A detailed and synthetic analysis of existing knowledge was performed, and insights into their future path in Industry 4.0 environments were provided as part of the contributions of this paper. The article also appraises initiatives in these domains, along with a succinct technology readiness analysis. To sum up, this study highlights MMs’ pivotal role in Industry 4.0, encompassing their influence on adaptability, automation, and efficiency.

## 1. Introduction

Modern industrial production has a growing requirement for flexibility since it allows companies to respond to shifting consumer demands, developing technologies, and new regulations [1]. This increases the expectations placed on the design and adaptability of robotics systems now and in the future. In order to meet this need, the literature has investigated prospective generations of transformable solutions for automation and logistics for small and large manufacturing systems. The robotics sector has undergone a transformation throughout the industrial age. Beginning with Robotics 1.0, where the primary goal was to provide early motorization to reduce repetitive human tasks, the field branched off with the integration of vision and feedback systems in Robotics 2.0. The transition to Robotics 3.0 represented a digital transformation that emphasized data exchange, human–machine collaboration, and the principles of Industry 4.0. The current era, Robotics 4.0, supports cloud-based controls, artificial intelligence, and IoT integration, and it aims for more organic interactions and comprehensive collaborative experiences. In parallel with these developments, a variety of robotic systems have emerged, including robotic arms for precision manufacturing, aerial drones, humanoid robots, autonomous vehicles, underwater explorers, surgical robots, and swarm robots [2]. Robotic systems known as mobile manipulators (MMs) have become a creative way to improve manufacturing flexibility and efficiency by combining the mobility of a mobile robot with the ability to manipulate objects through robotic arms. These systems can carry out a variety of activities with a high degree of flexibility and accuracy due to the integration of mobility and manipulation.

### 1.1. MMs: A History and Related Works

Basically, MMs consist of one or multiple robotic arms mounted on the top of a mobile platform. The system is surely equipped with proper sensors, actuators, and tools, enabling the MM to move according to surroundings safely and autonomously and to perform a task based on the selected tool for the end-effector of the arm. MMs can be designed for special functionalities by adapting their size, shape, payload, mobility, tools, and other characteristics. They are designed to work in special environments that can be on the ground (indoor or outdoor, for smooth or turbulent rout), aerial, or underwater. They can be designed to move sensitive small objects or huge ones for big constructions. Mobile platforms can be legged or wheeled. Wheeled is more common, which can be omnidirectional, holonomic, or nonholonomic. MMs gradually established their position for numerous applications, such as in agriculture, military, nuclear, space, mine, healthcare and assistive, domestic, search and rescue, and industrial fields. The primary focus of this article is on the industrial applications; however, some of the solutions in other areas have been addressed within some of the projects that are discussed as follows.

**Figure 1:**
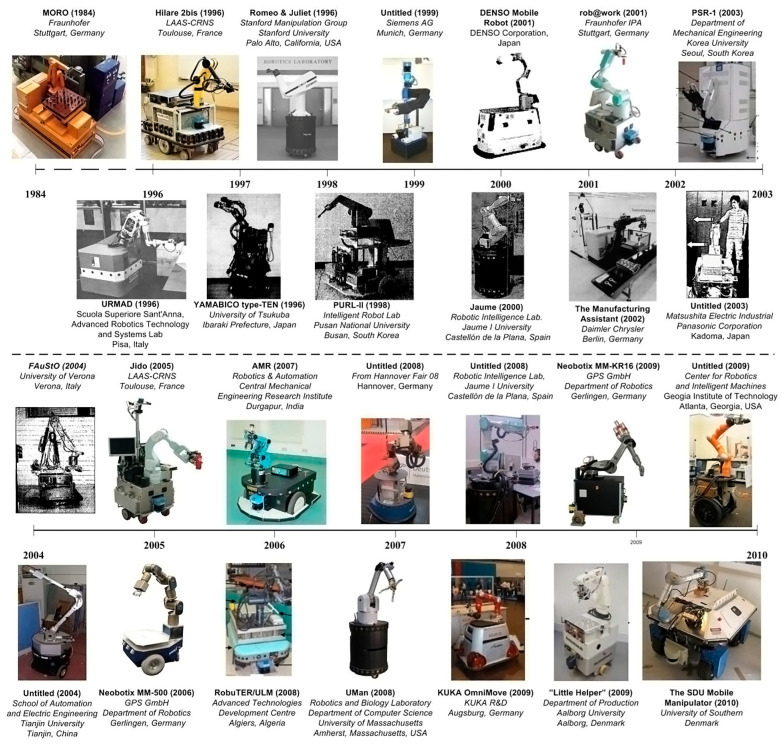
Timeline of existing MMs from 1984 to 2010 [3].

S. Bøgh et al. [3] provided an overview of existing MMs from the first prototype in 1984 (MORO) until 2010 (Figure 1). Nevertheless, the origins of MM creation may be traced back even farther to the Stanford Research Institute, which developed SHAKEY from 1966 to 1972 [4].

The journey of MMs has evolved through various stages, with the literature punctuating each transformative phase. The inception of MMs on production lines primarily aimed to bolster navigation, control, and perception, a trend significantly discussed in the RoboCup competitions [5]. As mobile platforms were integrated with serial manipulator arms, systems started to offer over nine degrees of freedom, paving the way for applications beyond the industrial sector, including service and rehabilitation [5].

However, extending the workspace of these manipulators posed both control and mechatronic design challenges [6]. The collaborative approach emerged as a promising avenue, with MMs being visualized as collaborative entities in industrial applications. This collaborative design, termed as Collaborative Mobile Industrial Manipulator, showcased an intricate blend of hardware, ranging from end-effectors to sensors, and software that incorporated planners and controllers [7]. Their versatility found interest in sectors such as logistics and manufacturing, even as research continued to optimize their interaction dynamics and autonomous controls.

Another groundbreaking direction was the convergence of unmanned aerial vehicles (UAVs) with MMs, hinting at the future of cooperative assembly and dynamic logistics [8]. Yet, the combined potential of UAVs and MRMs remains a nascent field, echoing the need for more focused investigations.

While these reviews provided insights into the challenges of system design [5], collaborative aspects [6], hardware-software architecture [7], and UAV-MM integration [8], there appear to be gaps in addressing certain facets of MMs.

In light of the above, our review aims to build, upon these foundations, a fresh perspective on the yet unexplored or under-represented areas, ensuring a more holistic understanding of the MM domain.

### 1.2. Towards Smarter Factory

By incorporating cutting-edge technologies, such as the Internet of Things (IoT), artificial intelligence (AI), advanced sensors, and robots into production processes, the fourth industrial revolution, Industry 4.0, seeks to develop smart factories that are highly efficient, adaptable, and responsive to market changes [9]. Due to the advent and development of Industry 4.0, robotic technology, which provided an essential contribution to the contemporary industry, has undergone significant growth in recent years. In order to satisfy the dynamic requirements of the factory of the future within the context of Industry 4.0, it is projected that the next generation of robots and its accompanying technologies will play a major role. Specifically, effectively utilizing the latest sensor technology is a crucial aspect of recent advancements in robotics. These advanced sensors not only enhance robots’ understanding of their surroundings but also play a vital role in ensuring safe and efficient communication between humans and robots. This domain stands out as one of the most significant areas driving progress in the field of robotic systems [10].

Introducing a new product for use in 4.0 environments requires considering the minimum requirements of Industry 4.0. Industry 4.0, by proposing a decentralized architecture instead of the traditional computer-integrated manufacturing (CIM) structure and introducing CPSs, enables close communication and data exchange among all components in real time [11]. As a result, for example, the ability of the device to communicate in the physical and cyber space is one of the necessary prerequisites. On the other hand, the capacity to comprehend and infer the environment, which is made possible by a variety of sensors and can be improved using AI approaches, is crucial for having a flexible robotic system such as MMs that can adapt effectively in changing and dynamic contexts. Therefore, MM developers are making progress and researching in order to prepare them as much as possible to find their way to real environments according to the current demands of industries. TRL is an approach to realize the degree of readiness of a concept for implementation under real conditions. Looking at MMs from a TRL perspective will assist in evaluating their suitability to integrate into 4.0 manufacturing systems.

### 1.3. Problem Statement

With the rapid evolution and adoption of Industry 4.0, there is a mounting emphasis on operational flexibility and adaptability in manufacturing environments. MMs have emerged as pivotal assets in addressing this demand, offering unique capabilities that transcend traditional robotics. However, while MMs inherently possess characteristics suitable for the Industry 4.0 paradigm, the issue of their operational and structural integration into a digitized production system remains. To fully harness the potential of MMs in Industry 4.0, it is imperative to identify and address areas where they must evolve. This review delves into the advancements necessary for MMs, ensuring their seamless and efficient deployment within the transformative landscape of Industry 4.0.

### 1.4. Motivation and Contribution

The motivation behind this review stems from the growing need to integrate agile, efficient, and adaptable robotic systems within modern factories. In the middle of the huge transition to Industry 4.0, understanding and leveraging the potential of MMs becomes vital. The aim of this review is to provide a comprehensive overview of MMs, their trajectory in the industry, and a roadmap for their future development. Our contribution lies in a meticulous analysis, a synthesis of existing knowledge, and insights into their future direction in Industry 4.0 environments.

### 1.5. Organization of the Paper

The rest of this article is organized as follows: This article investigates the application of MMs in manufacturing environments. Section 2 outlines the methodology the authors adopted for this review. A history of MMs and their application, employment, and integration into the industry are provided in Section 3. Section 4 proposes a taxonomy of several MM development areas required by Industry 4.0 and surveys the literature for efforts to progress in each area. Section 5 discusses the relationship between different development areas and analyzes different MM solutions from the vision of TRL, and finally, the paper is concluded in Section 6.

## 2. Methodology

To systematically review the developments of MMs in the context of Industry 4.0, our methodology was constructed in a multi-staged manner:

1. Preliminary Search Strategy

The research began by querying key databases, such as Scopus, IEEE, Google Scholar, and ScienceDirect, employing specific search strings. These strings contained terms such as “Mobile Manipulators”, “Industrial Applications”, “Industry 4.0”, “Robotics”, and “Automation”.

2. Segregated Literature Review

From our initial search, the literature falls into two categories:

- Industry 4.0-focused: These articles predominantly discussed the core concepts and facets of Industry 4.0 but might not explicitly mention MMs.
- Mobile Manipulator-focused: Papers that discussed the evolution, functionalities, and challenges of MMs, potentially without linking them directly to Industry 4.0.

3. Analysis of MM Applications in Industry

For the studies that delved into MMs, a key area of focus was understanding their applications across various industrial sectors. This involved categorizing the sectors where MMs have been implemented, such as manufacturing, logistics, assembly, and more; detailing the specific roles and tasks assigned to MMs in each sector; and highlighting the successes and challenges faced in each application.

4. Synthesis of Industry 4.0 Recommendations for MMs

From the Industry 4.0-centric literature, the principal areas that point to potential directions for MMs have been filtered. This exploration helped bridge the gap between Industry 4.0 aspirations and concrete MM developments.

5. Exploration of MMs

MM-centric papers were reviewed from historical and emerging challenges to chart their progression through time.

6. MMs in the Context of Industry 4.0

After separate investigations, an integrated analysis to understand how MMs are reshaped by Industry 4.0 paradigms was performed. This requires finding research that directly linked MMs with Industry 4.0, assessing the compatibility of current MM plans with Industry 4.0 criteria, and identifying areas where MMs may need more innovation to align with Industry 4.0 guidelines.

The effectiveness of this approach, compared to other methodologies, is to identify gaps related to the widespread adoption of MMs in Industry 4.0 and highlight areas that need more research.

## 3. Integration and Implementation of MMs in Industrial Environments

**Figure 2:**
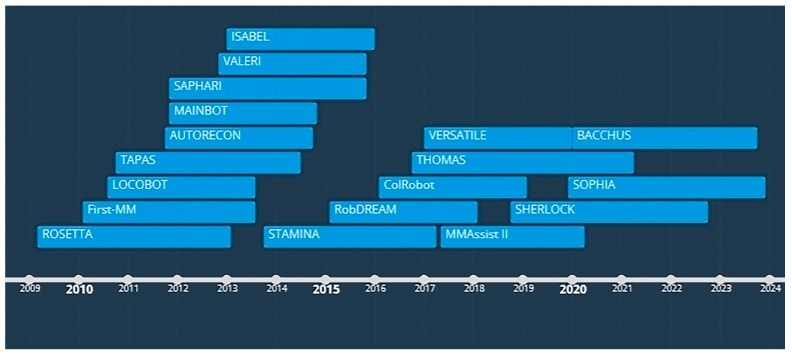
A timeline of different projects that contributed to MM development.

After the brief history of MMs, in this section, their journey from research to industry is presented. Then, the industrial application of MMs in real-world situations is reviewed and summarized at the end of Section 3.2. The overview presented in Figure 1 is followed by an introduction to more recent projects (Figure 2) that have contributed to the progress of MMs since 2010, with the findings of each mapped and reviewed in related sections.

### 3.1. From Research to Industry

Especially today, there is a close relationship between scientific research and the needs of industries. Sometimes, the needs of the industry cause the creation of projects for scientific investigation and find innovative ways and the feasibility of each of them. Sometimes, progress in science leads to finding solutions that were not even thought of before, while they help to improve the condition of factories. However, companies are more conservative in employing novel solutions due to a variety of factors, including significant investment costs, the need to modify an existing and successful system, etc. A crucial step in making MMs more prepared for serious entrance and widespread adoption in industries is the integration and implementation of MMs in laboratory settings or real production systems.

**Figure 3:**
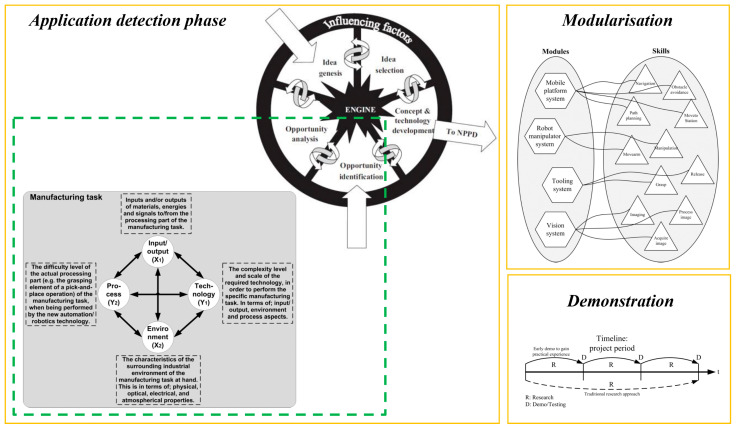
A representation of how to achieve proper infrastructure for MM integration inspired by [3].

S. Bøgh et al. [3] suggested some elements as the infrastructure of integrating MMs into the manufacturing systems. They believe that finding a common language between academy and industry is a vital infrastructure. New concept development (NCD) is an approach used to achieve it. Regarding this model, based on the main strategical objectives, different opportunities can be identified and analyzed to help select the best out of various ideas that address each opportunity. Then, the concept and development of the required technology can be achieved. They suggested a technology push manufacturing technology (TPMT) model to help identify potential opportunities and their analysis. These methodologies can lead us to find the best applications for new technologies, such as MMs. After this application finding, it is suggested to define modules and skills for MMs because a modular system can provide more flexibility. To showcase the practical applications of our selected technologies in real-world settings, we recommend employing a breakdown approach, wherein smaller demonstrations are conducted instead of a single comprehensive demonstration that may take an extended period. This approach allows for a more focused and targeted display of our technologies’ capabilities, enabling our audience to better understand their potential benefits in specific contexts. A representation of how to achieve proper infrastructure for MM integration is shown in Figure 3.

### 3.2. Industrial Applications

Similar to traditional industrial robots, MMs often find use in boring, stupid, filthy, and/or hazardous applications, with the added advantages of increased operational flexibility, expanded workspace, and enhanced overall equipment effectiveness. S. Bøgh et al. [3] investigated MMs as industrial assistance and classified their applications into logistics, assistive, and service applications (Table 1).

**Figure 4:**
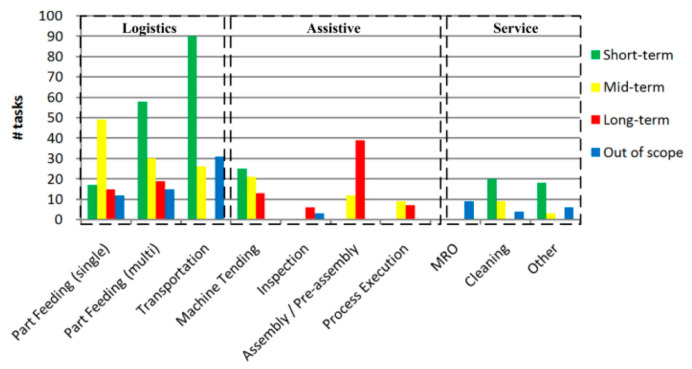
Suitability of utilizing MMs for each class of industrial operations.

The results of their analysis of MM suitability (in their case LHs) for 566 manual manufacturing tasks from five factories are presented in Figure 4, which shows that logistics, specifically transportation and part feeding, have the highest potential for the immediate implementation of MM technology.

**Figure 5:**
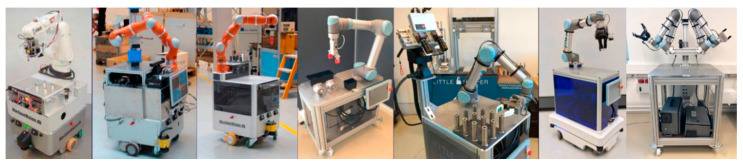
Little helpers from the first generation to the seventh one (from left to right) adopted from [12].

**Figure 6:**
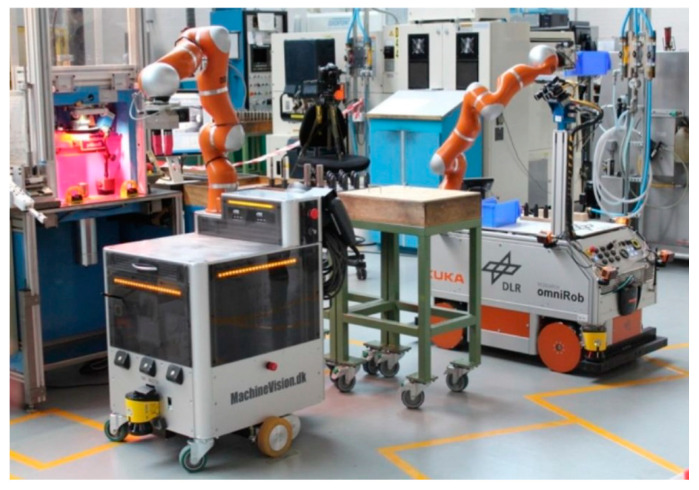
Little Helper 3 from Aalborg University (**left**); omniRob from KUKA (**right**).

**Figure 7:**
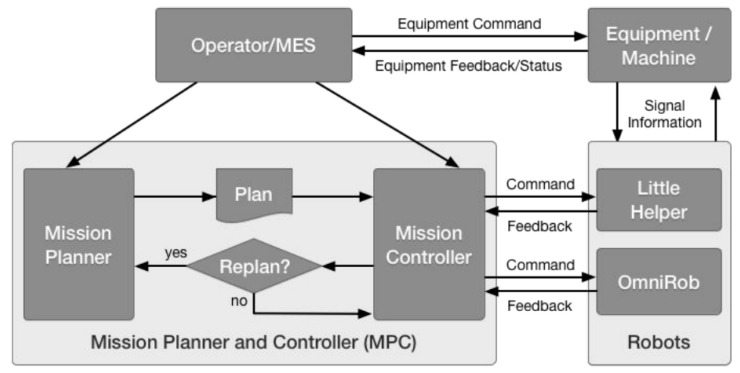
The controlling system proposed by [14] for integrating MMs into production systems.

For industrial settings, one of the known MMs, called Little Helper (LH), has seven different versions [12] and was developed at Aalborg University (Figure 5). The project TAPAS included real-world experiments with MMs, such as LH and omniRob, to assess the technology’s readiness for industrial application and their scheduling problems [13]. S. Bøgh et al. [14] believed that MMs are finding their way to more assistive tasks instead of the traditional logistic ones. Thus, they integrated two different types of MMs (LH 3 and omniRob) to collaborate with each other. LH takes part in the assembling operation of rotors, and the omniRob is deployed for logistics (Figure 6) at Grundfos. The duty of scheduling and task planning of the MMs is up to a central mission planner and controller (MPC). The important considerations are cycle time, travelling time of robots (however, they found it hard to detect only the travelling time; thus, they measured a combined average time), their capacity, and manufacturing goals. On the other hand, the environmental information is provided by MES as an input to the MPC (Figure 7). For their experiments, the main initial steps they defined are listed as follows: setting up MMs, setting up and verifying communications, adjusting the workstation, setting up the navigation, programming, and testing. Most of the errors for both MMs were announced to be communication failures and manipulation tasks, and their most serious challenges were in safety, integration with the existing production system, communication problems, and lack of error handling after losing connection.

O. Madsen et al. [15] also covered the simultaneous implementation of two MMs to assist in SQFlex pump production at Grundfos. They relied on skill-based programming and employed the same mission planner system for the parallel navigation of both MMs but without mentioning MES presence. Performance, user friendliness, and economic efficiency were the criteria to help them find the gap between current solutions and demands of the users. This resulted in a cycle time of more than 20 times longer than that observed for humans, as well as a total downtime of 58 min in a half work day due to navigation, assembly process, and communication errors. They decided that the reason for the long cycle time was related to limitations in its scenario, utilization, dexterity, and ability to perform simultaneous quality control. The main challenges they faced were safety, robustness and cycle time, and the reconfigurability and flexibility of MMs and the environment.

I. Nielsen et al. [16] proposed a methodology to implement MMs into cloud manufacturing flexible environments. Their methodology started with the design of MMs. Flexibility, modularity, standardization, autonomy, and dynamic nature were the key indicators to choose the LH for their demonstration. Next, they suggested a multiple-part feeding application. Then, the scheduling problem of MMs was highlighted with a mathematical model in the concept of the Bartender problem. For MM control in the production environment, they also adopted MPC architecture (Figure 7). Finally, for the real-world demonstration, they evaluated their methodology at the CR 1-2-3 impeller production site.

**Figure 8:**
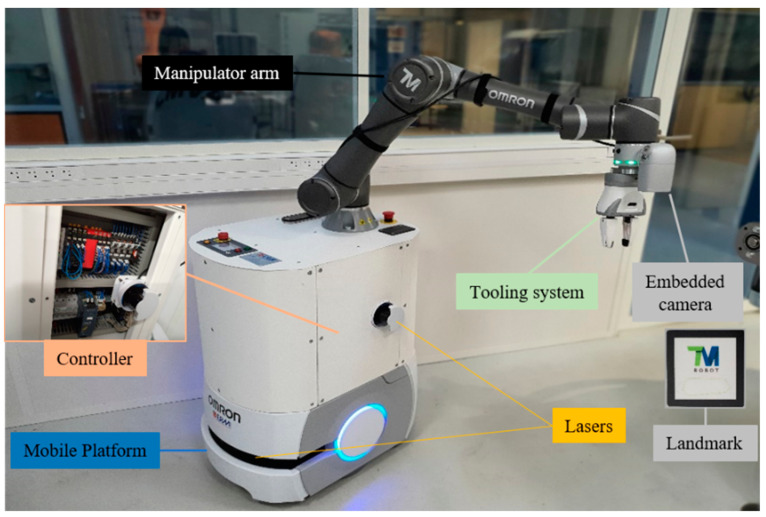
MOMA from OMRON adopted from [17].

Ghodsian et al. [17] suggested an approach for the successful integration of MMs into 4.0-based industries. They experimented with an MM from the Omron company called “MOMA” (Figure 8) at a laboratory-scaled production system that was supervised by MES [18] for automating the process of feeding raw material. R. Eckholdt Andersen et al. [19] described the implementation of an LH6 that performs part feeding application with a skill-based system (SBS) at the FESTO cyber-physical factory. Iterative design was used to create the LH6 system. The offset of the mobile platform led to the robotic arm’s imprecision, which was countered via a calibration technique. The module provided TCP connection between SBS and the PLC, and two new abilities for calling and listening were developed for seamless integration with SBS. Five different aspects of a part-feeding task were evaluated, and after 25 test runs with nine failures, it was determined that communication between the modules was the main execution bottleneck, and the mobile platform’s errors were caused by navigational errors around a corner close to the target locations.

**Figure 9:**
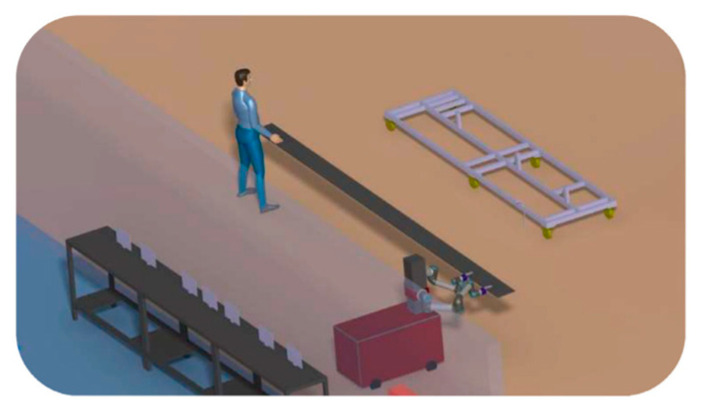
Using MM to carry large parts with human cooperation adopted from [21].

One of the other MM applications is concerned with the placement and co-manipulation of big pieces on tooling for precise procedures. According to the SOFITEC pilot case of the Sherlock project [20], even though the carbon fiber components of today are not very heavy, a single person cannot manipulate them due to their vast size (up to 7 m) and the distance between workstations. For its deployment, two people are often required, while one person positions the remaining instruments. The remaining employees only assist in following the leader and supporting the weight of the part, with only one operator adding value to the positioning process. The operators clearly work together to complete the assignment, employing force, visual, and speech modes to coordinate their actions. The use of dual-arm MMs for the placement and co-manipulation of massive composite aeronautics parts on precise process tooling has been investigated by [21] (Figure 9).

Another scenario within the Sherlock project concentrates on the assembly line of solar thermal collectors at CALPAK [22]. The operator must put together various thermal collector variations that range in size and weight. The operator must have two-side accessibility while lifting and moving the bulky thermal collectors around to complete the installation. Throughout a shift, these taxing duties must be completed repeatedly. Together with a higher level of exhaustion, chronic pain issues, such hand tendonitis, and back difficulties are also produced for human workers. Sherlock proposed the usage of a high payload collaborative MM. As the operator focuses on small assembly tasks, the high payload robot manages the large payload. AI-enabled onboard sensing devices keep an eye on the operator’s movements, assuring appropriate assembly by examining the order of the various processes. Moreover, augmented reality (AR) visualization tools that show the operator what steps to perform next in the assembling process are helpful.

**Figure 10:**
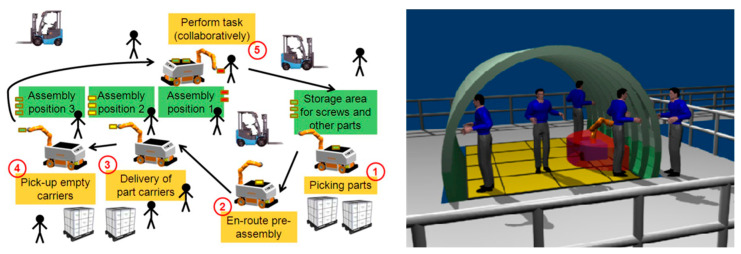
SAPHARI project: industrial applications required at KUKA (**left**) and aircraft assembly requirement of Airbus (**right**) adopted from [24].

The assembly of hydraulic pumps and the assistance of manual gas metal arc welding by use of the MM rob@work are two applications that are discussed in [23]. Figure 10 presents the MM applications of logistics and assembly of the KUKA manufacturing system (left) and collaborative assembly at airbus (right) within the project SAPHARI [24].

**Figure 11:**
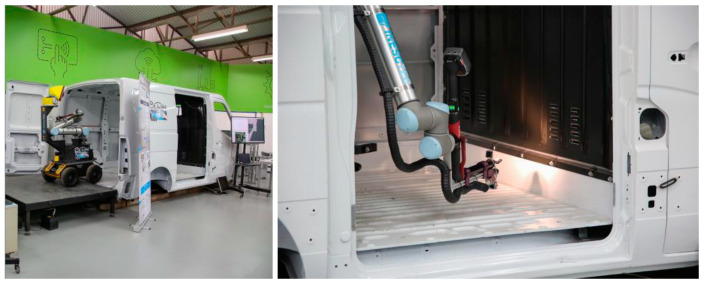
ColRobot prototype for automotive industries adopted from [25].

The ColRobot project integrates advanced robot technology with the needs of users in assembly processes. By using an MM as a “third hand”, the system can deliver kits, tools, and parts and hold work pieces while the operator performs their tasks collaboratively with the robot. Two cases from the automotive [25] and aerospace [26] industries in real-world operating settings were implemented and validated within this project. A ColRobot prototype (KMR IIWA) was presented and tested at ENSAM in [27] for kitting operations of THALES ALENIA SPACE France (TAS-F). Figure 11 shows the ColRobot prototype for fastener tightening and automatic refill in automotive industries [27,28].

BAZAR, a dual-arm MM, was developed at LIRMM (Laboratoire d’informatique, de robotique et de microélectronique de Montpellier) in 2016. It contains two KUKA LWR4+ arms on the top of a Neobotix MPO 700 omni-directional mobile platform. The VERSATILE EU project [29] aimed to investigate the use of MMs in three industry driven applications, the assembly of vehicle dashboards at PSA, the assembly of aircraft wing parts at AIRBUS, and the handling and packaging of shaver handles at BIC. The BAZAR’s introduction and its assistance in a smart logistic use case were presented in [30].

Fetch-and-carry operations in work spaces and settings shared with humans were investigated in the collaborative ISABEL (Innovative Autonomous and Intuitively Operated Service Robot for Efficient Handling and Logistics) project, which is financed by the Federal Ministry of Education and Research. The abilities to load and remove parts from machines, as well as autonomously choose and place items at stations, are priorities. In particular, “life science automation” and “semiconductor manufacturing” are the applications for which this is being performed. At Infineon, the ISABEL robot has undergone successful testing in a production setting. Wafer boxes could be effectively picked up, transported, and fed to equipment in the clean room [31].

**Figure 12:**
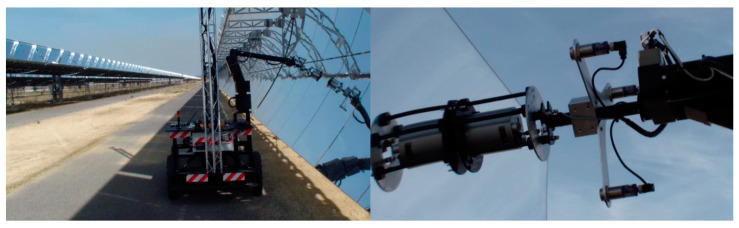
Mirror reflectivity measurement at the VALLE energy plant by MAINBOT adopted from [34].

Due to its versatility in performing a variety of duties, such as inspection, monitoring, and repair, MMs can find their way into industrial maintenance. In this sector, some applications are required regarding the MAINBOT project, including ubiquity sensing, leakage detection, and surface and interior equipment monitoring [32]. The MAINBOT project involves creating applications for service robots to independently carry out inspection activities on machinery that is organized either horizontally (using ground robots) or vertically (climbing robots) in large industrial plants. In order to achieve the project’s industrial goals, MAINBOT aims to use already-existing robots to implement creative solutions. Specifically, it wants to provide a way to measure various physical parameters at various locations using autonomous robots that can navigate and climb structures, handle sensors, or use specialized non-destructive testing equipment [33]. The strategy used by MAINBOT to accomplish scientific and industrial goals entails analyzing maintenance and inspection needs in large industrial plants, choosing inspection operations based on various criteria, designing modifications in existing MMs to meet the identified requirements, developing and adjusting inspection technologies to be integrated into autonomous MMs, justifying in simulation, integrating robotic platforms and non-destroyable materials, integrating these elements into new and existing robots, and verifying them using a mock-up and, at the end, in real-world case studies. One of these case studies that was carried out at the VALLE energy plant is shown in Figure 12. The tool containing the sensor was positioned perpendicular to the mirror in the actual operation, and the approach maneuver was carried out until the sensor hit the mirror. The toolholder’s three ultrasonic sensors produced signals that were utilized to regulate the trajectory.

IAAM (Institute for Applied Automation and Mechatronics) provides a few multidisciplinary projects that combine applied research with real-world application. FiberRadar is one of them, in which the MM OMNIVIL was used. A quality control technique is being developed for the production of high-strength fiber composite materials as part of the FiberRadar research project. Prior to casting, the approach displays the fiber structure in three dimensions to show any abnormalities and improve the stacking and flow of the fibers. To enable more precise quality control than is now achievable, the IAAM is in charge of creating a transportable measurement system for large-scale components used in the production of wind turbines [35].

With the automobile sector as a possible user, the STAMINA (sustainable and reliable robotics for part handling in manufacturing) project entails creating a fleet of robots that can carry out bin-picking and kitting activities in human-populated settings. The manufacturing line speed, which for example is one vehicle per minute for their use case at PSA, determines the robots’ operating pace [36].

In industries such as aerospace and shipbuilding, where huge components are manufactured in fixed production units, the VALERI (Validation of Advanced, Collaborative Robotics for Industrial Applications) project explores the necessity for MMs. An MM operating alongside human co-workers is recommended in such circumstances, as specialized stationary robotic devices are not cost-effective. The systematic design of such an MM is described by K. Zhou et al. [37], together with hardware and software concerns for load balancing, efficiency, and safety. The two responsibilities of applying sealant and visual inspection in aerospace production are also covered in their article, and a graphical user interface that may be customized is provided for evaluating and validating the system design. It shows that the robot they used for the sealing operation consists of a KUKA lightweight arm mounted on a KUKA omniRob mobile platform. VALERI is a special design of an MM developed for the project of the same name. VALERI consists of a KUKA omniRob mobile platform (with four omnidirectional wheels) and a 7 DoF lightweight robot (KUKA LBR4) on a vertical lift that provides two extra DoFs with a linear axis and a rotational capability, coming to a total of 12 DOFs. Three case applications—sealant, inspection of the applied sealant, and the inspection of braided carbon fiber parts—were selected for VALERI to operate in the aerospace production systems. This project covered the safety issues, sensor technologies specifically designed for safety and interaction with MMs [38], sensor fusion techniques [39], as well as workspace monitoring [40], human–robot collaboration aspects such as the hand-guidance of redundant MMs [41], and control strategies and effective redundancy solutions [42] to perform multiple operations at once to take advantage of the whole robot system’s capabilities [43].

The industrial application of MMs in real-world situations is summarized in Table 2.

## 4. Industry 4.0: Requirements for Developing Adaptable MMs

Industry 4.0 has revolutionized production and industrial approaches focused on the application of advanced technologies such as AI, machine learning, and the IoT, which lead to a new era of automation and digitization. Robotics is no exception to this, and its development process has changed based on the needs of Industry 4.0. Thus, robots are no longer just tools for performing dangerous or repetitive tasks, but they are now being used in new and innovative ways to improve efficiency and productivity. Safety navigation, part separation and inspection expertise, adherence to safety standards, dependability, sustainability, and the capacity to be use-case independent and adjustable with little programming are the major needs for MMs. MMs must also be manageable by a resource management system for the business, and shop floor employees must be able to utilize and modify them instantly to new handling jobs.

In the following, a few ways in which Industry 4.0 has changed robots are categorized.

### 4.1. Increased Connectivity and Interoperability

One of the principals of Industry 4.0 is to connect system components in a decentralized architecture. Thanks to developments in IoT and M2M communication technologies, the connectivity of MMs in industrial environments has been enhanced. MMs can now conduct more complicated tasks and work together more effectively with other devices because of this improved connection.

Interoperability frameworks create a stable communication bridge among diverse IIoT devices, each with unique software and protocols. These span organizations, different vendors, and a wide range of cloud and fog/edge service providers with diverse architectures [44]. Viewed from an industrial point of view, interoperability provides a global standard that ensures a distinct data format, API, and communication protocol for data management, whether on local edge devices or cloud servers [45]. Therefore, the smart industry needs a universally accepted applicable standard that can effectively manage IIoT applications in industrial infrastructure. In the context of IoT, several standards and protocols have emerged to facilitate this connectivity and interoperability. A set of established protocols such as HTTP (Hypertext Transfer Protocol), MQTT (Message Queuing Telemetry Transport), and CoAP (Constrained Application Protocol) serve as the backbone for data communication. Notably, the OPC UA (Open Platform Communications Unified Architecture) standard plays a pivotal role in fostering interoperability across various industrial control systems [46]. MMs may more easily be integrated into current production processes thanks to these protocols, which support interoperability between various equipment and systems.

### 4.2. Data Collection and Analysis

Collecting data and its analysis to achieve useful information in real-time is one of the other important aspects highly recommended by Industry 4.0. Thus, MMs are equipped with a wide range of sensors and data collection tools to collect and transfer data in various required metrics to managerial data storage. Advanced analytics and machine learning algorithms can be used to optimize the performance of MM itself and also to discover the surrounding environment. Gathering and analyzing large amounts of data in real-time, assisting MMs to adapt to changing and dynamic conditions, and optimize their performance are all performed to improve efficiency and productivity.

Utilizing AI methodologies to discover the patterns and trends helped to predict potential issues before occurring. Predictive maintenance is one of the advantages of this analysis. At high levels, complete autonomy is expected based on the wide range of data transfers from all of the system devices. In such systems, self-optimization and decision-making tools are strengthened.

### 4.3. Improved Flexibility

Flexibility refers to the ability of the system to adapt quickly to the changes in conditions, demands, and requirements. Industry 4.0 is characterized by the use of advanced technologies to create more flexible and adaptable manufacturing systems. Industry 4.0 expects MMs to operate flexibly and to flexibly integrate into the production system, so that any changes in the whole system could be perceived by the MM and it can react to it dynamically. To address this issue, research has been conducted.

#### 4.3.1. Flexible Navigation, Path Planning, Localization, and Mapping

The distinction between preprogramming and real-time programming of the common planning algorithms depends on whether the environment map is known or unknown. Many studies based on structured environments have been conducted in the past, which suggests that CMIM might accomplish localization and navigation by preprogramming. Industry 4.0’s trend has evolved into a more adaptable mechanism, allowing the system to update the map concurrently and therefore improve efficiency. SLAM, or simultaneous localization and mapping, is a popular method for structuring the world at the same time. In tandem with SLAM’s real-time mapping, vision-based line tracking, an element of the “Guided” driving approach, offers an intermediate degree of autonomy. This technique allows mobile robots to follow predetermined paths using visual cues, especially in environments with set structures [47]. Campbell et al. [48] detailed several robot localization methods, including GPS (satellite-based), odometry (wheel rotation measurement), inertial navigation systems (INS, without initial reference but prone to errors), and map-based location.

Motion planning is difficult due to MMs’ distinctive features. Planning algorithms should take into account the behavior of the system to provide optimum plans quickly. Preparing separately for the mobile base and manipulator might result in inferior plans. It has not been thoroughly investigated how to coordinate movements between the mobile base and manipulator during planning, but applying machine learning approaches to determine the MM’s capabilities can assist. System dynamics are not heavily used in the planning algorithms used today; instead, they concentrate on designing geometric paths [49]. The innovative MM (MRP) was developed for flexible manufacturing systems that consider design, navigation, and docking aspects by [50] within the THOMAS project.

Path planning refers to the task of identifying a safe and efficient route from the present position of a vehicle to a target destination while avoiding any obstacles [51]. It is a vital problem, especially according to the control engineers [52]. The mobile robot path planning can be split into two categories of global and local path planning. Global path planning refers to the path generation in a known environment, while local path planning addresses an unknown environment with unexpected moving obstacles that can be observed by the robot’s sensors [53]. A. Montazeri et al. [52] suggested the following four important criteria for a path planning algorithm: optimization, completeness, accuracy, and execution time. G. Spyros [54] also classified the path planning methods into three categories of reactive control, representational world modeling, and their combination. F. Wayne et al. [55] formulated an optimization problem for MMs by separating the degrees of freedom (DOF) for mobility and for the manipulator. They developed a simulated annealing method for solving their model. M. Zhao et al. [56], on the other hand, applied a genetic algorithm. Path planning for a welding MM was considered in the research of W. S. Yoo et al. [57]. For the purpose of controlling an MM with two arms in path tracking and point-to-point motion, M. H. Korayem et al. [58] studied the use of a path planning algorithm in the feedback linearization approach. F. Burget et al. [59] offered a BI2RRT (bidirectional informed rapidly exploring random tree) planning methodology for creating asymptotically optimum pathways for MMs under task restrictions. A* algorithm has been used for the MM’s end effector path planning problem by G. Silva et al. [60]. For a class of hyper-redundant mobile soft manipulators that approach and handle objects while simultaneously avoiding obstacles based on potential field of velocity, S. Mbakop et al. [61] proposed a curvature control technique. Their method was built on the sliding mode concept, which converges in a finite amount of time.

A flexible stochastic approach was provided by M. Haddad et al. [62] for the point-to-point trajectory planning of nonholonomic wheeled MMs moving in a structured workspace while considering geometry, kinematics, and dynamic constraints. In order to assure base motion coordination along the predetermined path while generating the best motion trajectory for the end-effector, extra kinematic constraints and generalized coordinates were chosen in the approach of M. Habibnejad Korayem et al. [63]. The approach for creating a suboptimal trajectory for an MM under mechanical and control restrictions was presented in the study of G. Pajak and I. Pajak [64]. The path of the end-effector was described as a curve that may be parameterized by any scaling parameter. Control constraints, collision avoidance requirements, and limitations related to the existence of mechanical restrictions for a certain manipulator configuration were taken into consideration. The research of M. Giftthaler et al. [65] is concerned with the challenge of kinematic trajectory planning for MMs under holonomic operational-space tracking and non-holonomic constraints. They utilized a constrained SLQ for their work and established experiments for a 26 DoF mobile platform. I. Akli [66] introduced a manipulability percentage index (MPI) in their research to avoid singularity and to place the mobile robot in areas that guarantee regular access.

#### 4.3.2. Flexible Scheduling

The use of a decision-making framework for integrated task scheduling and resource mobility planning in hybrid production systems is discussed in [67]. The modular robot operating system (ROS) framework, an open-source platform that enables the interfacing of multiple robot kinds and robot brands, serves as the foundation for the proposed design, making it open. As a result, it is simple to include new sorts of resources in the decision-making process. The framework supports the scheduling of operations for the assembly of various components by interpreting various assembly sequences using a unified digital world modeling methodology. Using the mobile robot behavior as an assessment measure, such as execution time and travel distance, the system is able to optimize the task plans that are created. The implemented system also offers a number of improvements, including the ability to reconfigure the production system by moving mobile resource platforms to various workstations according to needs, the ability to generate the most practical and efficient robot motion plans, and the ability to enable online access to real-time shopfloor status using the identified obstacles as input. The suggested approach offers a viable response to the problems in hybrid production systems and is suited to small- to medium-sized production systems. Future research should, however, concentrate on improving and fine-tuning the suggested system in order to make it usable for a wider-scale application. The system should be integrated with a factory’s legacy system in this approach. This will make the proposed decision-making framework more practical and relevant to a larger range of production systems by enabling seamless communication between the existing systems and it.

A novel scheduling issue involving a single MM performing feeding duties in a production cell was investigated in [13]. Mission planners must choose the best feeding sequence to reduce the overall traveling time of the MM while taking into consideration the unique characteristics of the robot and a variety of technological limitations in order to complete all tasks within the permitted limit of battery capacity. With the purpose of locating the best solution, a novel mix-integer linear programming model was created. To demonstrate the effectiveness of the suggested methodology, a specific real-world example of an impeller production line composed of four feeders was detailed. The outcome was very appropriately used in actual feeding operations, and it showed that all feeders had no trouble feeding components to the production line. By taking into account a high number of feeders and/or a lengthy planning horizon, the complexity of the issue classified as being NP-hard rises. As a result, a meta-heuristic approach was used to resolve the large-scale mobile robot scheduling problem. Additionally, a rescheduling system based on collected schedules and input from the shop floor should be created to address real-time disruptions.

#### 4.3.3. Flexible Control and Coordination

Control systems include a number of components, such as control type, method, and controller, with the goal of improving the performance of the entire system in terms of tracking, disturbance rejection, resilience, and other factors. In general, there are three different ways to control CMIM: manually, semi-autonomously, and totally autonomously. Manual control, a secure but complex method of system control, has received a lot of attention in studies to this point. Both manual control and autonomous control are benefits of the semi-autonomous control technique. The greatest method to create a completely intelligent industry is through autonomous control, which enables the system to learn and respond autonomously. Table 3 shows the differences between the three control types presented by [5].

According to [68], it can be dangerous to utilize a completely autonomous MM for some industrial tasks, such as material handling and machine tending, since an incorrect representation of the working environment could lead to costly machinery being harmed. In contrast, using a completely teleoperated MM could take a lot of operator time. For this reason, they designed a semi-autonomous MM to perform machine tending duties under human supervision safely and effectively. Using the high-level task description, the robot may create motion plans and provide simulation results to a human for approval. The human operator has the option of giving the robot permission to carry out the automatically created plan or giving the planner more information to improve the plan. The person can choose to use teleoperation to securely complete the activity if the workspace model contains areas with a high level of uncertainty.

Although MMs can be partially pre-programmed and semi-autonomous, the variety of industrial environments may make it difficult for the system to be used in various situations. Additionally, the system’s intelligence at this point is insufficiently advanced to handle independently some issues or unclear data. The future of MMs would be fully autonomous systems, allowing the system to carry out entire tasks on its own. This may increase the maturity of systems in terms of the fourth industrial revolution, but the intelligent system has to be reliable and safe in addition to being able to spot mistakes in both organized and unstructured situations.

In order to create flexible manufacturing systems, research within the project RedRobCo suggested employing a force torque sensor close to the end effector to implement code-free programming and natural hand guiding. Using haptic feedback, the control structure manages kinematic redundant arm configurations and steers clear of them. The article contains laboratory experiments on the MM chimera to show the functionality of the controller design, along with a thorough study of potential singularities [69]. The interest in contactless MM control in delicate settings, such as clean rooms, is discussed within the project CapSize. The study in [70] described a capacitive sensing-based contactless control system that is resistant to mechanical impact, dirt, occlusions, and poor lighting. The sensor can be integrated into a workspace or mounted directly on a robot arm. To recognize gestures, a straightforward model-based method is utilized, making it simple to adapt to diverse geometric restrictions. The article showed the system’s capabilities by regulating an MM’s end-effector velocity in a 3D task space and giving the operator feedback. Additionally, in the CollRob project, Ref. [71] discussed how to program robot systems entirely without writing a single line of code, and it presents a practical method for programming delicate MMs with hand guiding that makes use of a force torque sensor that is mounted near the end effector. The results of laboratory tests were reported, and a detailed explanation of the suggested control method was provided.

### 4.4. Sensing and Perception

One of the first prerequisites of MMs to work in smart manufacturing systems is the ability to perceive its surroundings and accordingly make decisions. A wide variety of sensors and capturers were developed and are under development. The use of new technologies to improve the perception of MMs is addressed in much of the research.

The first stage for MMs to perceive their surroundings is to determine where they are in relation to their surrounding world. The difficulty in using the calibration technique on MMs is brought on by the variation in error between the mobile platform and stationary manipulators. The eye-in-hand calibration approach was used in the publication [72] using an MM to identify the actual coordinates of objects in the world’s coordinates.

The advancement of hardware (GPUs) and deep learning techniques over the past ten years has greatly benefited the challenging discipline of computer vision known as object detection. With algorithms such as face detectors functioning in many common applications, object detection is increasingly becoming the state-of-the-art AI technique in our modern world. Broadly speaking, object detection is a technique used for locating an object of interest in a photo and determining if it is present. G. Andrianakos et al. [73] addressed employing object detection to monitor assembly-line personnel using machine learning methods. When an assembly task is finished, the system should signal that information. There are two techniques used to determine this. As pieces need to be placed together in order to be assembled, the first one is comparing the locations of the assembly parts (PD). Detecting which object is grasped by a human is the other method (HPD). As the phrase says, it may be difficult to detect for micro parts, while it may be effective for bigger parts. In order to ensure that the proper assembly work is being carried out and to avoid mistakes, the article suggested employing a two-level check technique utilizing the PD and HPD methodologies. The research also emphasized how critical it is to establish a threshold for grabbing likelihood in order to prevent false alarms and the potential advantages of adopting error detection techniques in the industry to speed up production, cut waste, and enhance product quality.

The BACCHUS project (MoBile Robotic PlAtforms for ACtive InspeCtion and Harvesting in AgricUltural AreaS) investigated the use of dual-arm MMs in agricultural areas for active inspection and selective harvesting. Within this project, for effective grasping, I. Sarantopoulos et al. [74,75] proposed approaches to detect the desired object in a cluttered environment and to make enough space around it for easier picking by pushing the surrounding objects. Instant segmentation was investigated by I. Kleitsiotis et al. [76].

### 4.5. Human–Robot Interaction and Safety

Collaborative robots (cobots) are one significant way that Industry 4.0 has impacted HRI for MMs. Cobots are made to work alongside people, as opposed to traditional robots, which are made to operate without the assistance of humans. Because employees can easily direct the cobot’s motions or even physically engage with it when necessary, HRI has become more natural and intuitive. With the increased interaction between humans and robots, safety has become an even more critical concern. Industry 4.0 has also brought about advancements in safety technology for MMs. For example, some MMs are now equipped with sensors and cameras that can detect and avoid obstacles, as well as software that can predict potential collisions and stop the robot before it occurs.

Depending on the use of the robotic arm, certain criteria must be applied to MMs: (1) The robot arm rests when the mobile platform is moving: The robot arm may be viewed as a load, and the risk assessment for the whole danger must be determined using an analogy to normal mobile robot standards. The precise description of a secure place in which the robot will be carried is essential. Robotic standards must be taken into account for the interfaces on which the report completes its duty. (2) The robot arm performs assembly or processing operations while in motion: The required risk assessment must be conducted for the robot arm using all robotic standards [77]. Following that, the entire application, including the moving platform, robot arm, and surrounding area, must be assessed in accordance with EN ISO 10218 part 2, which addresses the integration of industrial robot applications [78].

A variety of AI-based tools are being developed by researchers so that robots can interact with and comprehend humans more effectively. N. Dimitropoulos et al. [21] categorized these tools into five main classes. (1) The “process perception module” is one of these tools, which makes use of computer vision to recognize the tasks being carried out by human workers. This module can assist the robot in comprehending what the user is doing and modifying its behavior as necessary. (2) The “workstation monitoring module” is another instrument that employs sensors to find impediments and human employees in the workspace. The robot’s motion planners then receive these data and utilize them to create a safe path and prevent collisions. (3) In order to provide the robot with a more thorough grasp of its surroundings, the “shop floor digital representation module” builds a digital version of the workspace. (4) The “task and action planner” aids in workload distribution between human and automated workers by determining the most effective course of action for each activity. (5) Finally, “autonomous learning strategies” allow the robot to modify its behavior in response to input from human workers, making sure it is satisfying their wants and preferences. By combining their efforts, these tools can make it easier for humans and robots to operate together safely and effectively, increasing productivity and lowering the risk of accidents.

Machine learning techniques were used in the ColRobot project to validate workplace monitoring and human identification for flexible safety [79].

Discovering the inefficiencies in hybrid human–robot collaborative workstations and improving them by AI-based solutions and smart mechatronics is one of the remarkable issues addressed by the SHERLOCK project [21]. In this project, several articles focused on HRC [80,81,82,83], such as the work on dual-arm co-manipulation architecture with enhanced communication [84], psychological and physiological acceptance of robots by humans [85], and the use of AI and wearable devices for collaborative assembly [86,87,88]. The project also included using human activity recognition sensors to improve the performance of collaborative MMs [89,90], using machine learning [73], and developing a task and action planning approach in human–robot collaborative cells using AI [91]. HRC for picking and moving common objects with humans was discussed in [92]. The study in [93] reviewed the use of a digital twin (DT) for HRC enhancement.

**Figure 13:**
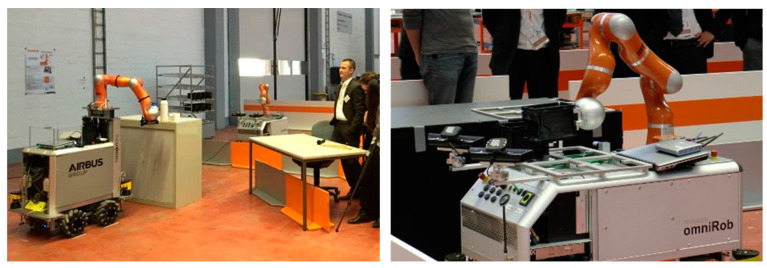
SAPHARI demonstrations: (**left**) Airbus use case, (**right**) Kuka use case adopted from [95].

Safety and HRI are primary goals of the SAPHARI (Safe and Autonomous Physical Human-Aware Robot Interaction) project. A collision-free robot motion was planned, and the robot constantly responded to unexpected dangerous situations by adapting its planned motion, slowing down, or stopping. This was accomplished by continuously monitoring the robot’s environment using multiple depth sensors to gather information about obstacles and predict their motions. These techniques have been put into practice on an omniRob that can pick up, position, and deliver things to people in a shared workspace while enhancing the route and collision avoidance behavior for both the robot platform and manipulator [94]. The project concentrated on two industrial use cases, developed by the industrial partners AIRBUS (Figure 13-left) and KUKA (Figure 13-right), that clearly included intentional physical interaction between a human and a robot coworker. Under the direction of DLR, a professional service scenario for hospitals was designed, including close daily interactions between medical professionals and an aiding robot [95].

SOPHIA (Socio-physical Interaction Skills for Cooperative Human–Robot Systems in Agile Production) mainly aimed to address the problem of HRC. E. Lamon et al. [96] suggested an interface for MMs that enables them to communicate safely while adhering to human commands, utilizing haptic admittance and visual tracking modules. Safety was also addressed in [97], which presented MOCA-S, an MM with a capacitive tactile cover for secure in-person HRI.

**Figure 14:**
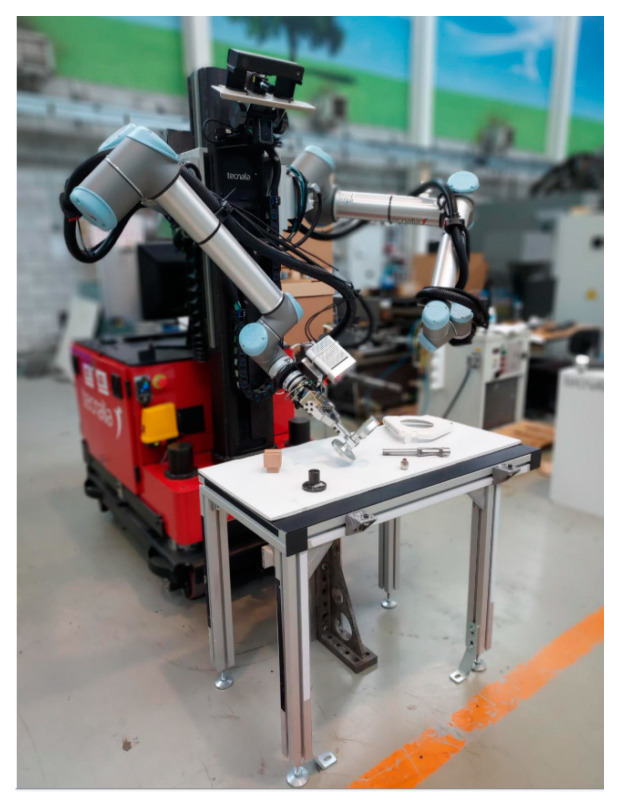
MM designed in the THOMAS project adopted from [105].

By using mobile dual-arm robots that can sense their surroundings and collaborate with other production resources, including human operators, the THOMAS project sought to build a reconfigurable shop floor. The project’s goals included enabling movement on resources and goods, enabling perception of the job and surroundings, dynamic workload balancing, enabling quick programming and task execution, and ensuring secure HRC. The research areas within this project can be categorized into five main areas of focus. Firstly, they introduced an innovative MM presented in Figure 14 [50] and their application in assembly systems [98], and they enhanced the HRI and HRC by introducing wearable devices such as smartwatches [99] and by employing AR-based software [100,101]. The development of a digital world model [102] and DTs for designing and optimizing the design was investigated in [103,104].

OMNIVIL has a self-designed mobile platform with holonomic kinematics for agility in dynamic and unstructured environments. It has three main advancements, one of which is a redundant workspace monitoring system that employs thermal and RGB cameras, lidar sensors, and deep convolutional neural networks to identify and categorize barriers and human employees. The scalable, adaptable zone-based navigation paradigm uses case-based behavior to enhance HRI [106].

### 4.6. Virtualization

Industry 4.0 enables virtualization for MMs by utilizing various technologies, such as AR and virtual reality (VR). In order to remotely operate MMs and see the manipulator’s activities in real time, these technologies may be utilized to construct virtual representations of the environment and the items in it. DTs also enable systems to optimize product design, improve production processes, and reduce maintenance costs.

Industry 4.0 expects MMs to provide enhanced visualization capabilities, enabling operators to have a better understanding of the task at hand and allowing for the more precise manipulation of objects. This includes the ability to visualize the manipulator’s workspace, detect and track objects, and display augmented information on the environment. Additionally, MMs are expected to be integrated with other Industry 4.0 technologies, such as the Industrial Internet of Things (IIoT), to enable data collection and analysis to optimize performance and productivity. Overall, Industry 4.0 expects MMs to provide a more efficient and effective means of completing tasks, with the help of advanced visualization technologies.

In order to combine and update the virtual environment with real-time data from several modules, including damper, barriers, and person detection, Ref. [103] implemented a DT infrastructure for a flexible assembly line. In order to evaluate HRI through gesture recognition, a scenario was put up in the GAZEBO physics simulation engine and included CAD files, virtual controllers, laser scanner data, simulated stereo camera data, and actual Kinect data.

In order to enable the dynamic behavior of HRC reconfigurable systems, Ref. [104] presented a DT-based system to complete the loop between the design and operation phases. To create the system’s layout and work plans, the DT was combined with an AI decision-making logic. The physical assets and unified interfaces of the DT smart models are dynamically updated during runtime, creating a real-time planning scenario that is utilized when re-design is required. The suggested method aims to get past current restrictions on execution sequencing and the online reconfiguration of hybrid industrial systems. Their DT model provided the data required to optimize the system’s structure by allocating duties to MMs and human operators at various workstations. A group of perception modules are deployed during execution to keep an eye on the execution, the surroundings, and people’s behavior using a variety of sensors. These sensors record pictures in both 2D and 3D. These data are synthesized using the applicable DT data structures and are used to populate the real-time planning scenario as an activity map.

To benefit from the advantages of DT, it is necessary to address the issues with CPS and IoT as well as improvements in simulation environments with physics engines. To create more precise DTs, improved modeling of complicated jobs is also required. Better data flow between the DT’s many modules is also crucial. Key enablers in this respect include further advancements in semiconductor processing and communication technology [93].

In line with Industry 4.0, a contemporary production approach known as “cloud manufacturing” (CMfg) turns traditional manufacturing resources into services by utilizing cloud computing, the IoT, and virtualization, and then it makes them accessible through the Internet. When necessary, the employment of the various manufacturing services may be cost-optimized thanks to an intelligent, cloud-based platform that controls them. The idea of CMfg encompasses every stage of a product’s life cycle, from creation and design through manufacture and testing to maintenance while in use. Manufacturing as a service (MaaS) is the term for the cloud-based provision of production resources, such as hardware and software programs, as well as knowledge-based competences. The best services are chosen, capacities are established, and orders are planned and regulated using CAD data and databases of knowledge-based machines and materials. In this approach, a large number of clients may best access a wide range of providers (even those that are locally distributed), plan their own control and management layer (cloud layer), and manage production capacity, manufacturing processes, operations, and transactions.

The ability of MMs to interact and communicate has developed to the point that they could potentially be included in the overall production network. The aforementioned benefits enable MMs to operate efficiently and effectively in environments for advanced manufacturing, particularly CMfg, which naturally followed the introduction and success of cloud computing. In CMfg, manufacturing resources (MMs) and manufacturing capability (task flexibility and robotic mobility) are offered for requests in distinct sites within factories through centralized management. Moreover, these benefits of MMs help CMfg manufacturing lines, which are intended to be transitory in nature, produce small batches, while also allowing for longer production runs. As a result, the MM technology has enormous promise for the manufacturing sector as a whole and CMfg in particular [16].

### 4.7. Cyber Security

Cybersecurity is a critical aspect of Industry 4.0, and MMs must comply with industrial cybersecurity standards and regulations to ensure the security and integrity of production processes.

According to Industry 4.0, MMs should have a robust cybersecurity posture to resist against possible threats, including data leaks, hacker attacks, and other malicious activities. To transfer sensitive data to other equipment and systems on the shop floor, such as project specifications, production plans, and other confidential information, and in order to avoid unwanted access to the system, MMs must have secure communication channels.

The security requirements of MMs can be categorized into physical-based and network-based attacks [107]. Network interfaces and unsecured USB ports need to be avoided since they can be used to install malware or steal data. In order to request new jobs and connect with other machines and people, the robot has to be able to engage with external systems such as MES. To guard against unapproved access or data breaches, this communication should be secure. The robot should have safeguards in place to thwart attempts at manipulation by users and operators during secure contact. This covers both the robot’s secure initial commissioning and the secure reprogramming by shop floor staff.

An architecture for MM security was developed and integrated into the CHIMERA in [107]. With only designated channels connecting the subsystems, their security architecture for the CHIMERA robot was built on subsystem isolation. Additionally, they divided the software stack among many pieces of physical equipment, making it more difficult for attackers to breach other subsystems. They deployed AppArmor2 on the devices to provide precise permission for users and processes. An obligatory access control system called AppArmor enables the isolation of certain apps in terms of access to other resources (such as files and devices). They employed it to restrict the alteration of vital system components while yet granting system integrators limited access to the internals of the CHIMERA.

## 5. Discussion

**Figure 15:**
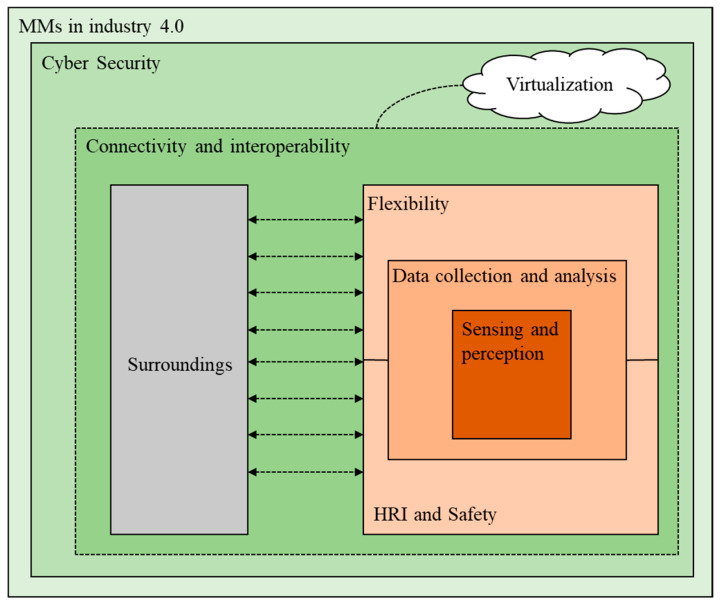
Semantic link between the key principles to consider for their integration into Industry 4.0-based manufacturing systems.

Hence, a variety of technologies and tools have been established or are being developed in response to the needs and expectations of Industry 4.0 for it to be more efficient and simpler for MMs to be properly integrated in smart production systems. Figure 15 summarizes the tight semantic link between the elements discussed in the preceding section, and the following provides an example to illustrate it.

As was mentioned in the previous section, MMs’ collaboration has drawn particular interest due to their characteristics, and ensuring safety is one of the most crucial conditions of this collaboration. This safety refers to both the MM itself and all the components of the MM environment, such as humans or other supporting machinery and equipment. Many tools are utilized in the field of mobile robots or robotic arms that build an appropriate platform for their connection with people in order to establish safety for the collaboration of MM with humans. On the other hand, several flexible control mechanisms for MMs are recommended. Guidance systems with human hands are one of the control strategies that promote human safety. Nevertheless, in order to use such a technique, the MM must be capable of interpreting its environment in order to identify movements. To do this, MM has to be fitted with sufficient sensors that will allow it to collect and analyze data. The capacity to communicate and interact with its surroundings, including sensors, other machines, and central control systems, is the primary requirement for MMs of this generation.

NASA, the US Department of Defense, and other organizations frequently utilize the technology readiness level (TRL) methodology to determine technological maturity. The TRL is based on a scale of 1 to 9, with 9 being the most advanced technology. The usage of TRLs provides universal, consistent discussions of technological maturity across various technology kinds. The TRL begins at phase one, when the technology is in its concept, and ends at level nine, where it is more developed and has undergone testing and launch. With the use of this technique, management may quickly assess the technology’s maturity and make choices on its development and adoption. It is not a standalone tool; rather, it is one of a number of tools required to monitor the progression of research, development, and testing initiatives inside an organization. This tool is used by investors and international funding organizations to determine the project category that best meets their goals.

**Figure 16:**
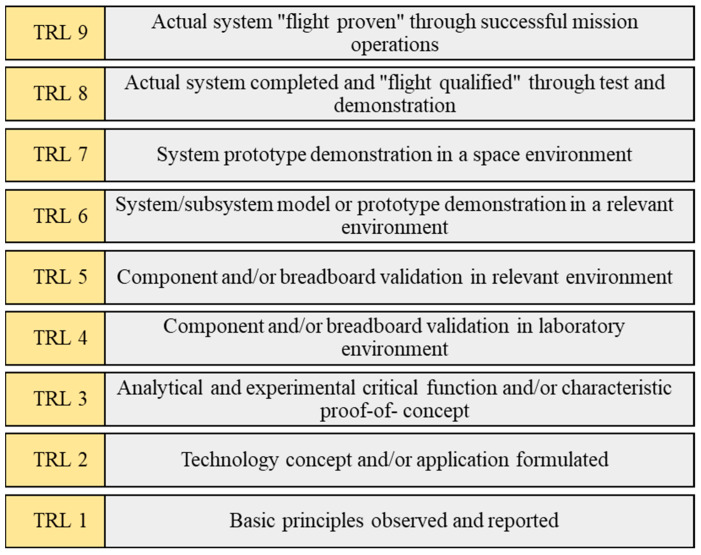
Technology readiness levels.

Phases one through three of TRL (TRL1: basic principles observed, TRL 2: technology concept formulated, TRL 3: experimental proof of concept) are regarded as the idea levels. The essential notion has been researched, the foundational research has been completed, and a proof of concept has been created. The technology has been developed and authorized in a laboratory or in another pertinent setting, and is now in TRL stages four and five at the prototype level (TRL 4: technology validated in a laboratory, TRL 5: technology validated in a relevant environment). The technology reaches the validation stage in TRL phases six and seven, with functional demonstrations taking place in a pertinent operating setting. The production level is involved in the final TRL phases eight and nine (Figure 16). The technology has been finished, authorized, and successfully tested in real-world settings.

Several studies have been carried out using competition, simulation, or laboratory tests. Real industrial environments, on the other hand, are more complicated and include more people and performance objectives. It is well recognized that it is quite challenging to simulate and utilize the actual industrial environment. Some cutting-edge MMs should be tested in real settings since lab and simulation settings differ greatly from actual circumstances. It would be more practical for the researchers to test the system, receive realistic input, and then progress their research based on it. Mobility, manipulation skills, and the integration of organizing and perception systems have all undergone major advancements. MMs are employed in applications such as logistics, industrial support, warehouse automation, and research. In order to solve issues with robustness, adaptation to dynamic situations, and human–robot interaction, businesses and research institutes are actively developing and enhancing technology. Future developments in machine learning, AI, and sensor technologies are anticipated to substantially improve the performance and capabilities of MMs.

While the focus of this review was specifically on MMs, advancements in the general field of mobile robots and robotic arms were not exhaustively considered. Given the interconnected nature of these fields, some innovations and findings from mobile robots could be applicable to MMs, and these might not have been comprehensively addressed in our review. However, potential applications of the findings for industries aiming to integrate or improve their automation processes use insights related to MM developments. This enables a more informed adoption of automation solutions compatible with Industry 4.0 principles. On the other hand, the identified gaps and emerging trends in MMs can guide researchers and developers in tailoring their future projects, ensuring that the next generation of MMs is better aligned with Industry 4.0 requirements.

## 6. Conclusions

The ability to respond quickly to customer demands and increase productivity without excessive costs is important for manufacturing in the context of flexibility, which is an essential feature of smart and competitive factories. Emerging technologies, such as cloud operations and industrial artificial intelligence, in the Industry 4.0 era can facilitate the development of flexible production systems. Robotic systems and mobile robots have shown a key role in improving the efficiency and autonomy of manufacturing systems. Combining robotic arm manipulation capabilities with mobile robot mobility allows for the creation of mobile manipulators, which may be employed for a variety of applications to increase the flexibility and effectiveness of the system as a whole. Even though MMs are used in various contexts, in this article, we reviewed their industrial applications. They are especially well suited for intralogistics tasks, such as part feeding inside assembly lines and transportation, as most of the articles investigated. Inspection, maintenance, or other shopfloor operations such as welding, screwing, painting, and spraying are some of the other applications, which are mentioned in the literature. However, these must be modified to meet the new innovative concepts and demands of the manufacturers, as knowledge is progressing.

The developing concept of Industry 4.0 entails combining several technologies, including AI, CPS, cloud computing, IoT, etc., to create smarter factories for the future. Because they provide the production process autonomy and enable it to self-configure, self-supervise, and self-heal, smart factories are essential to Industry 4.0. By enabling devices and services to interact autonomously to fulfill production objectives, CPS is another significant technology used to add autonomy to the production process. IoT is utilized as an integration layer to support autonomous processes for coordination, cooperation, and collaboration, whereas system integration enables cutting-edge technologies to cooperate to boost the manufacturing process’s autonomy. Big data analysis and contemporary human–computer interactions are also essential to Industry 4.0 since they address the variety of actors and data, respectively, and enable transparent human integration into the manufacturing process.

Assessing the design and implementation challenges in MM systems revealed that AI has the potential to advance this topic. In general, AI is a branch of cognitive science with active research programs in fields including robotics, machine learning, and image processing. Mobile robots have been enhanced both at the device and system levels by the techniques and knowledge that have been produced. AI techniques have advanced mobile robot navigation to autonomous driving and obstacle avoidance, which have been investigated a lot in the literature. However, there is a lack of information about MMs with the ability to function in cloud settings at the system level, which can offer on-demand computing services and assistance for rational decision-making throughout the scheduling process with MMs. Thus, MM technology has the potential to help industrial processes become more flexible and productive. The use of autonomous mobile robots (AMRs) for interior positioning and navigation has been made possible by recent advancements in AI and computer capacity. AMRs are more flexible and adaptable than conventional AGVs since they function autonomously and with decentralized decision-making; thus, their utilization as the base of MMs is more common, although there are different types of AMRs. As they are more compact and agile, they can integrate into workplaces more effectively. This increases manufacturing flexibility and assists them to fulfill the needs of the current production process. MMs with AMR platforms can operate alongside people as robot coworkers and as an assistive system.

In this regard, one of the main topics of discussion is safety and communication with humans. For example, wearables technologies such as AR enable people to interact with MMs in a more intelligent way, increasing collaboration and coordination among them to enhance production processes, decrease waiting times, and increase safety and cost efficiency, all of which are important characteristics of Industry 4.0. In general, virtualization and the use of digital models, simulations, and digital twins are essential for MMs. These new improvements enhance the need for MMs to be equipped with cybersecurity systems. The main and fundamental field of achieving all the mentioned features depends on the installation and use of suitable sensors and how to receive data from them, as well as communication with other components to access and exchange information and analyze data instantly.

This research not only deepens our understanding of MMs within the realm of Industry 4.0 but also sets the stage for their more profound societal impacts. As manufacturing processes are made more adaptable and efficient, a faster response to market demands can be facilitated by industries, potentially leading to a reduction in resource wastage and the fostering of more sustainable production cycles. Furthermore, with the enhanced human–robot interaction facilitated by MMs, a more inclusive work environment is anticipated, where human tasks are complemented by machines, paving the way for new job roles and opportunities.

As a relatively new and growing technology, MMs need more improvements to enter and establish a foothold in production systems on a large scale. However, the most important thing to achieve such a goal is extensive experimental tests at the level of real systems that are rarely mentioned. The basic challenges in the few who have mentioned this issue are safety and HRI, communication stability, robustness, communication, and battery. This is why this technology is still in TRL 6 or 7, except for special cases in terms of readiness level. Consequently, it is crucial to concentrate more on the battery and energy issues, as well as the stability and dependability of the technologies utilized on MMs in future research in order to really deploy them.

## Figures and Tables

**Table 1:** Classification of MM industrial applications.

Mobile Manipulators Applications		
Assistive Tasks	Logistics Tasks	Service Tasks
Machine tending	Transportation	Maintenance, Repair and overhaul (MRO)
Assembly/Pre-assembly	Part feeding (multi)	Cleaning
Inspection	Part feeding (Single)	
Process execution		

**Table 2:** A summary of industrial applications of MMs.

Citation	Application	Industry	Project	MM	Year
[3]	Multiple-part feeding	Grundfos		Little Helper	2011
[14]	Assembly, machine tending, quality control	Grundfos	TAPAS	LH3 and omniRob	2014
[15]	Assembly, quality control, logistics	Grundfos	TAPAS	Two LHs	2015
[16]	Multiple-part feeding	CR 1-2-3 impeller line		LH	2017
[19]	Part feeding	Festo cyber-physical factory		LH6	2017
[21]	Placement of common object with human	Aerospace	SHERLOCK		2014
[22]	High payload assembly	CALPAK	SHERLOCK		2023
[23]	Assembly of hydraulic pumps and the assistance of manual gas metal arc welding	Hydraulic pumps	MORPHA	rob@work	2002
[24]	Logistics and assembly	Automotive, aerospace	SAPHARI	omniRob	2012
[27]	Kitting	TAS-F	ColRobot	KMR IIWA	2020
[28]	Fastener tightening and automatic refill	Automotive industries	ColRobot	ColRobot prototype	2020
[30]	Smart logistics, assembly of vehicle dashboards, assembly of aircraft wing parts, and handling and packaging of shaver handles	PSA, AIRBUS, BIC	VERSATILE	BAZAR	2019
[31]	Fetch-and-carry	Clean room	ISABEL	ISABEL	2023
[35]	Quality control	Production of wind turbines	FiberRadar	OMNIVIL	2021
[36]	Bin-picking, kitting	PSA	STAMINA		2014
[37]	Applying sealant and visual inspection	Aerospace, shipbuilding	VALERI	VALERI	2014
[32,33]	Industrial maintenance	VALLLE energy plant	MAINBOT	MAINBOT	2014, 2023

**Table 3:** A comparison of different MM control strategies adopted from [5].

System Control	Description	Benefits	Drawbacks
Manual	Fully controlled by humans	Timely error handling	Can be complicated
Semi-automatic	Partial human control	More robust	May be inefficient
Automatic	No human intervention	Efficient	Decision costs can be high

## Abbreviations

**Abbreviation**	**Description**
AI	Artificial Intelligence
AMR	Autonomous Mobile Robots
AR	Augmented Reality
CIM	Computer Integrated Manufacturing
CMfg	Cloud Manufacturing
cobots	Collaborative robots
CPS	Cyber-Physical Systems
DOF	Degrees of Freedom
DT	Digital Twin
IIoT	Industrial Internet of Things
IoT	Internet of Things
LH(s)	Little Helper(s)
MM(s)	Mobile Manipulator(s)
ROS	Robot Operating System
TRL	Technology Readiness Level
UAV(s)	Unmanned Aerial Vehicle(s)
VR	Virtual Reality
